# Triethyl­ammonium [2-eth­oxy­carbonyl-2-(2-methyl­benz­yl)-6,9-dinitro-3-oxo­bicyclo­[3.3.1]non-6-en-8-yl­idene]azinate

**DOI:** 10.1107/S1600536811018095

**Published:** 2011-05-20

**Authors:** Vaduganathan Manickkam, Doraisamyraja Kalaivani, S. Rajeswari

**Affiliations:** a PG and Research Department of Chemistry, Seethalakshmi Ramaswami College, Tiruchirappalli 620 002, Tamil Nadu, India; b Department of Chemistry, National Institute of Technology, Tiruchirappalli 620 015, Tamil Nadu, India

## Abstract

In the title salt, C_6_H_16_N^+^·C_20_H_20_N_3_O_9_^−^, the cations and anions are connected by N—H⋯O hydrogen bonds. The structure is consolidated by weak C—H⋯O inter­actions.

## Related literature

For general background to adducts containing a bicyclic [3.3.1]nonane skeleton and the synthesis of closely related compounds, see: Gnanadoss & Kalaivani (1985 ▶). For related structures, see: Balasubramani *et al.* (2011 ▶). For puckering parameters, see: Cremer & Pople (1975 ▶).
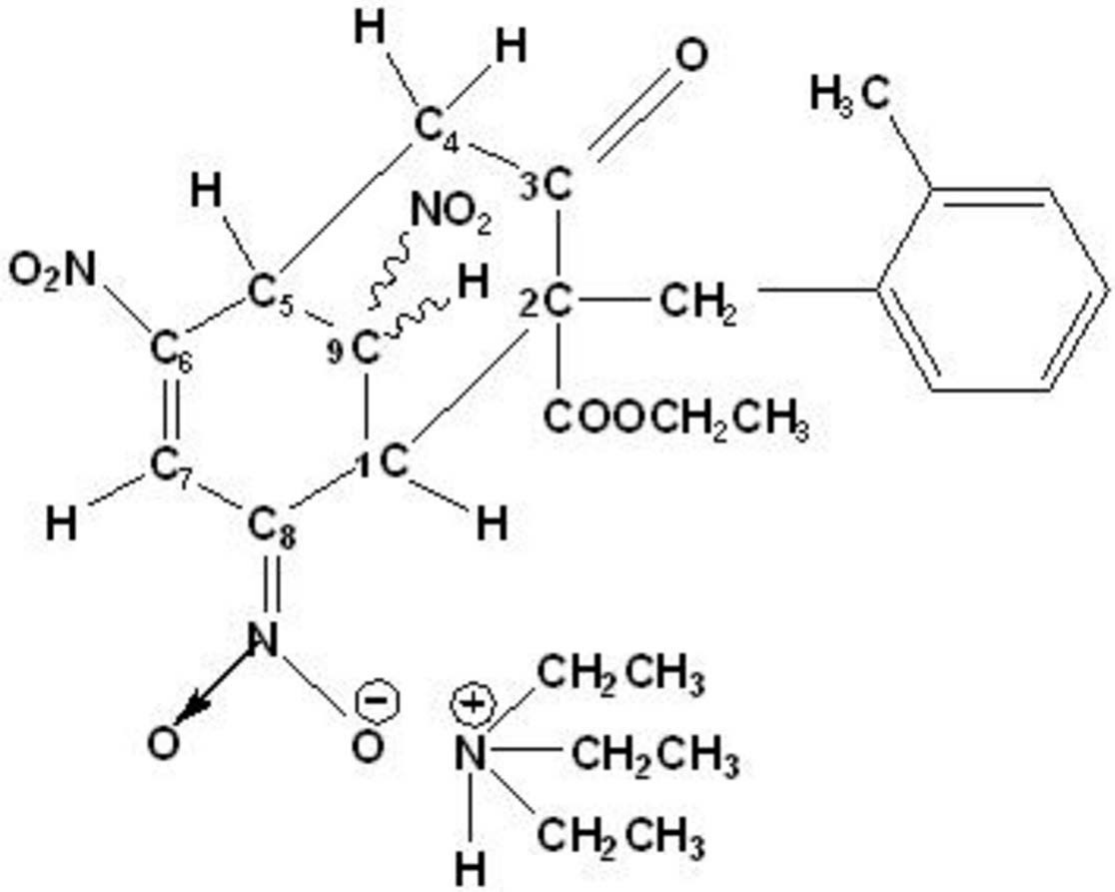


## Experimental

### 

#### Crystal data

C_6_H_16_N^+^·C_20_H_20_N_3_O_9_^−^
*M* *_r_* = 548.59
Triclinic, 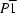

*a* = 8.2820 (3) Å
*b* = 10.9776 (4) Å
*c* = 15.9881 (6) Å
α = 97.103 (2)°
β = 100.991 (5)°
γ = 93.283 (2)°
*V* = 1411.08 (9) Å^3^
*Z* = 2
Mo *K*α radiation
μ = 0.10 mm^−1^
*T* = 293 K
0.30 × 0.25 × 0.20 mm

#### Data collection

Bruker Kappa APEXII CCD diffractometer
Absorption correction: multi-scan (*SADABS*; Bruker, 2004 ▶) *T*_min_ = 0.971, *T*_max_ = 0.981
25290 measured reflections
4754 independent reflections
3459 reflections with *I* > 2σ(*I*)
*R*_int_ = 0.027

#### Refinement

*R*[*F* ^2^ > 2σ(*F* ^2^)] = 0.054
*wR*(*F* ^2^) = 0.197
*S* = 1.07
4754 reflections
361 parameters
H atoms treated by a mixture of independent and constrained refinement
Δρ_max_ = 0.42 e Å^−3^
Δρ_min_ = −0.23 e Å^−3^

### 

Data collection: *APEX2* (Bruker, 2004 ▶); cell refinement: *SAINT-Plus* (Bruker, 2004 ▶); data reduction: *SAINT-Plus*; program(s) used to solve structure: *SIR92* (Altomare *et al.*, 1993) ▶; program(s) used to refine structure: *SHELXL97* (Sheldrick, 2008 ▶); molecular graphics: *ORTEP-3* (Farrugia, 1997 ▶) and *Mercury* (Macrae *et al.*, 2008 ▶); software used to prepare material for publication: *SHELXL97*.

## Supplementary Material

Crystal structure: contains datablocks global, I. DOI: 10.1107/S1600536811018095/pv2406sup1.cif

Structure factors: contains datablocks I. DOI: 10.1107/S1600536811018095/pv2406Isup2.hkl

Supplementary material file. DOI: 10.1107/S1600536811018095/pv2406Isup3.cml

Additional supplementary materials:  crystallographic information; 3D view; checkCIF report

## Figures and Tables

**Table 1:** Hydrogen-bond geometry (Å, °)

*D*—H⋯*A*	*D*—H	H⋯*A*	*D*⋯*A*	*D*—H⋯*A*
N4—H1*A*⋯O4^i^	0.97 (4)	1.78 (4)	2.740 (3)	168 (3)
C17—H17*A*⋯O7^ii^	0.96	2.55	3.434 (5)	154

Symmetry codes: (i) 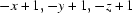; (ii) 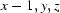.

**Fig. 1.**
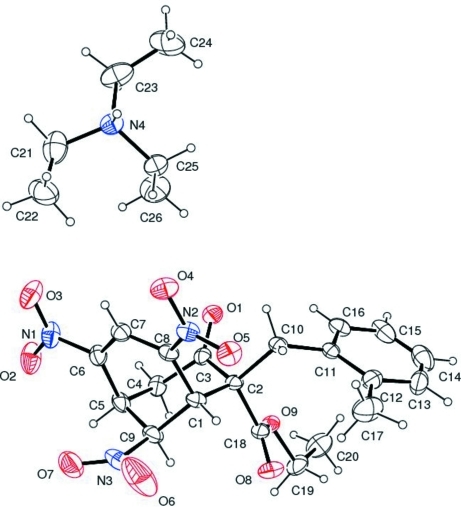
A view of the title compound showing the displacement ellipsoids plotted at 30% probability level.

**Fig. 2.**
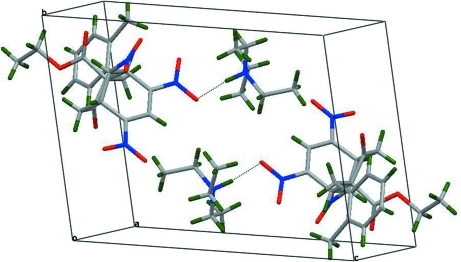
Unit cell packing of the title compound showing hydrogen bonds.

## supplementary crystallographic information

### Comment

A series of adducts containing bicycle [3.3.1]nonane skeleton, closely related to the title molecule, has been synthesized in our laboratory (Gnanadoss & Kalaivani, 1985). We have recently reported the structures of two such bicyclic molecules derived from 1,3,5-trinitrobenzene, ethyl 2-benzyl-3-oxobutanoate / ethyl 2(4-nitrophenylmethyl)-3-oxobutanoate and triethylamine (Balasubramani *et al.*, 2011). In this article we report the crystal structure of the title compound (Fig. 1) which is a bicyclic adduct derived from 1,3,5-trinitrobenzene, ethyl 2(2-methylphenylmethyl)-3-oxobutanoate and triethylamine. The values of puckering parameters (Cremer & Pople, 1975) of the six membered ring (C1/C2/C3/C4/C5/C9) atoms: *Q* = 0.501 (3) Å, θ = 52.6 (3)° and φ = 304.1 (4)°, imply that this ring has slightly distorted chair conformation. The puckering parameters of another six membered ring (C1/C8/C7/C6/C5/C9) with values, *Q* = 0.604 (3) Å, θ = 170.5 (3)° and φ = 119.4 (16)°, indicated that it has slightly distorted envelope conformation. The hydrogen bonding observed between the N–H group of triethylammonium cation and oxygen atom of  the nitronate ion (Tab. 1 & Fig. 2) may probably be the driving force for the extraordinary stability of the adduct.

### Experimental

A saturated ethanolic solution of 1,3,5-trinitrobenzene (2.1 g, 0.01 mol) was mixed with a saturated ethanolic solution of ethyl 2(2-methylphenylmethyl)-3-oxobutanoate (2.3 g, 0.01 mol). To this mixture triethylamine (6 ml) was added and shaken well for about two hours. The resulting maroon red coloured solution was kept as such for twenty four hours till the colour changed from maroon red to orange red. The orange solution was distilled under reduced pressure to get a viscous mass which was washed repeatedly with 100 ml of dry ether and redissolved in absolute alcohol (20 ml). To the alcoholic solution, 200 ml of dry ether was added and refrigerated between 273 - 283 K for 6 h to get the red orange crystals of the title compound (yield 60%). Single crystals were obtained from ethanol at room temperature by slow evaporation (m.p. 409 (2) K).

### Refinement

The hydrogen atom bound to the N atom waas located from a difference electron density map and allowed to refine freely. The rest of the hydrogen atoms  were identified from the difference electron density peak and were included in the refinement with the following constraints: C—H = 0.93, 0.97, 0.96 and 0.98Å for aromatic, methylene, methyl and methyne groups, respectively, and *U*_iso_(H) = 1.5*U*_eq_(methyl C) or 1.2*U*_eq_(the rest C/N atoms).

### Crystal data

**Table:**

C_6_H_16_N^+^·C_20_H_20_N_3_O_9_^−^	*Z* = 2
*M**_r_* = 548.59	*F*(000) = 584
Triclinic, *P*1	*D*_x_ = 1.291 Mg m^−^^3^
Hall symbol: -P 1	Mo *K*α radiation, λ = 0.71073 Å
*a* = 8.2820 (3) Å	Cell parameters from 7927 reflections
*b* = 10.9776 (4) Å	θ = 2.1–23.8°
*c* = 15.9881 (6) Å	µ = 0.10 mm^−^^1^
α = 97.103 (2)°	*T* = 293 K
β = 100.991 (5)°	Prism, red
γ = 93.283 (2)°	0.30 × 0.25 × 0.20 mm
*V* = 1411.08 (9) Å^3^	

### Data collection

**Table:**

Bruker Kappa APEXII CCD diffractometer	4754 independent reflections
Radiation source: fine-focus sealed tube	3459 reflections with *I* > 2σ(*I*)
graphite	*R*_int_ = 0.027
ω and φ scans	θ_max_ = 24.6°, θ_min_ = 1.3°
Absorption correction: multi-scan (*SADABS*; Bruker, 2004)	*h* = −9→6
*T*_min_ = 0.971, *T*_max_ = 0.981	*k* = −12→12
25290 measured reflections	*l* = −18→18

### Refinement

**Table:**

Refinement on *F*^2^	Primary atom site location: structure-invariant direct methods
Least-squares matrix: full	Secondary atom site location: difference Fourier map
*R*[*F*^2^ > 2σ(*F*^2^)] = 0.054	Hydrogen site location: inferred from neighbouring sites
*wR*(*F*^2^) = 0.197	H atoms treated by a mixture of independent and constrained refinement
*S* = 1.07	*w* = 1/[σ^2^(*F*_o_^2^) + (0.1121*P*)^2^ + 0.3791*P*] where *P* = (*F*_o_^2^ + 2*F*_c_^2^)/3
4754 reflections	(Δ/σ)_max_ < 0.001
361 parameters	Δρ_max_ = 0.42 e Å^−^^3^
0 restraints	Δρ_min_ = −0.23 e Å^−^^3^

### Special details

**Table:**

Geometry. All e.s.d.'s (except the e.s.d. in the dihedral angle between two l.s. planes) are estimated using the full covariance matrix. The cell e.s.d.'s are taken into account individually in the estimation of e.s.d.'s in distances, angles and torsion angles; correlations between e.s.d.'s in cell parameters are only used when they are defined by crystal symmetry. An approximate (isotropic) treatment of cell e.s.d.'s is used for estimating e.s.d.'s involving l.s. planes.
Refinement. Refinement of *F*^2^ against ALL reflections. The weighted *R*-factor *wR* and goodness of fit *S* are based on *F*^2^, conventional *R*-factors *R* are based on *F*, with *F* set to zero for negative *F*^2^. The threshold expression of *F*^2^ > σ(*F*^2^) is used only for calculating *R*-factors(gt) *etc*. and is not relevant to the choice of reflections for refinement. *R*-factors based on *F*^2^ are statistically about twice as large as those based on *F*, and *R*- factors based on ALL data will be even larger.

### Fractional atomic coordinates and isotropic or equivalent isotropic displacement parameters (Å^2^)

**Table:**

	*x*	*y*	*z*	*U*_iso_*/*U*_eq_	
C1	0.8740 (3)	0.2324 (2)	0.75355 (15)	0.0522 (6)	
H1	0.8218	0.1492	0.7321	0.063*	
C2	0.8230 (3)	0.27903 (19)	0.83970 (14)	0.0466 (5)	
C3	0.9196 (3)	0.4041 (2)	0.87753 (14)	0.0496 (5)	
C4	1.1035 (3)	0.4074 (3)	0.88464 (16)	0.0614 (7)	
H4A	1.1501	0.3579	0.9280	0.074*	
H4B	1.1517	0.4915	0.9024	0.074*	
C5	1.1458 (3)	0.3578 (3)	0.79762 (17)	0.0646 (7)	
H5	1.2655	0.3548	0.8043	0.078*	
C6	1.0857 (3)	0.4370 (3)	0.73069 (16)	0.0602 (6)	
C7	0.9348 (3)	0.4139 (2)	0.67895 (15)	0.0563 (6)	
H7	0.9016	0.4654	0.6380	0.068*	
C8	0.8283 (3)	0.3146 (2)	0.68587 (14)	0.0503 (5)	
C9	1.0620 (3)	0.2288 (3)	0.77050 (17)	0.0633 (7)	
H9	1.0942	0.1803	0.8176	0.076*	
C10	0.6325 (3)	0.2887 (2)	0.82428 (16)	0.0529 (6)	
H10A	0.6111	0.3723	0.8144	0.064*	
H10B	0.5819	0.2346	0.7721	0.064*	
C11	0.5488 (2)	0.2574 (2)	0.89530 (15)	0.0503 (6)	
C12	0.4672 (3)	0.1425 (2)	0.8923 (2)	0.0699 (8)	
C13	0.3932 (4)	0.1216 (4)	0.9618 (3)	0.1014 (13)	
H13	0.3374	0.0454	0.9611	0.122*	
C14	0.4015 (5)	0.2119 (6)	1.0311 (3)	0.1091 (14)	
H14	0.3526	0.1958	1.0767	0.131*	
C15	0.4793 (4)	0.3219 (4)	1.0328 (2)	0.0907 (10)	
H15	0.4843	0.3827	1.0794	0.109*	
C16	0.5510 (3)	0.3450 (3)	0.96667 (18)	0.0656 (7)	
H16	0.6040	0.4227	0.9688	0.079*	
C17	0.4587 (5)	0.0413 (3)	0.8189 (3)	0.1157 (14)	
H17A	0.4167	0.0711	0.7657	0.173*	
H17B	0.3869	−0.0270	0.8257	0.173*	
H17C	0.5672	0.0151	0.8184	0.173*	
C18	0.8679 (3)	0.1858 (2)	0.90228 (17)	0.0548 (6)	
C19	0.9359 (4)	0.1581 (3)	1.0482 (2)	0.0841 (9)	
H19A	0.8604	0.0844	1.0323	0.101*	
H19B	1.0477	0.1337	1.0534	0.101*	
C20	0.9144 (5)	0.2231 (4)	1.1295 (2)	0.1043 (12)	
H20A	0.9940	0.2931	1.1465	0.156*	
H20B	0.9297	0.1690	1.1726	0.156*	
H20C	0.8051	0.2503	1.1231	0.156*	
C21	0.7584 (5)	0.7677 (5)	0.5416 (3)	0.1150 (14)	
H21A	0.8417	0.7901	0.5937	0.138*	
H21B	0.7635	0.8324	0.5058	0.138*	
C22	0.7964 (7)	0.6475 (5)	0.4941 (3)	0.1389 (19)	
H22A	0.7931	0.5834	0.5296	0.208*	
H22B	0.9043	0.6572	0.4808	0.208*	
H22C	0.7158	0.6260	0.4417	0.208*	
C23	0.5420 (6)	0.8821 (3)	0.5943 (2)	0.1097 (14)	
H23A	0.5659	0.9386	0.5553	0.132*	
H23B	0.6075	0.9122	0.6509	0.132*	
C24	0.3634 (7)	0.8812 (5)	0.5984 (3)	0.1381 (18)	
H24A	0.2981	0.8418	0.5446	0.207*	
H24B	0.3345	0.9643	0.6093	0.207*	
H24C	0.3426	0.8367	0.6439	0.207*	
C25	0.5726 (4)	0.6660 (3)	0.62405 (19)	0.0764 (8)	
H25A	0.5936	0.5860	0.5969	0.092*	
H25B	0.4593	0.6606	0.6320	0.092*	
C26	0.6848 (6)	0.6938 (5)	0.7104 (2)	0.1209 (14)	
H26A	0.7975	0.6952	0.7035	0.181*	
H26B	0.6641	0.6314	0.7450	0.181*	
H26C	0.6647	0.7726	0.7381	0.181*	
N1	1.1922 (3)	0.5370 (3)	0.71987 (16)	0.0803 (7)	
N2	0.6879 (3)	0.2852 (2)	0.62465 (13)	0.0589 (5)	
N3	1.1120 (4)	0.1648 (3)	0.6904 (2)	0.0924 (9)	
N4	0.5911 (3)	0.7588 (2)	0.56488 (14)	0.0652 (6)	
O1	0.8513 (2)	0.49432 (16)	0.89576 (13)	0.0696 (5)	
O2	1.3326 (3)	0.5515 (3)	0.76464 (16)	0.1121 (9)	
O3	1.1441 (3)	0.6069 (2)	0.66769 (16)	0.1045 (8)	
O4	0.6478 (2)	0.35738 (18)	0.56828 (12)	0.0768 (6)	
O5	0.5978 (2)	0.18796 (18)	0.62164 (12)	0.0731 (5)	
O6	1.0281 (5)	0.0774 (4)	0.6518 (3)	0.186 (2)	
O7	1.2365 (4)	0.2025 (3)	0.67191 (19)	0.1193 (10)	
O8	0.8667 (3)	0.07679 (17)	0.88015 (14)	0.0816 (6)	
O9	0.9032 (2)	0.23944 (15)	0.98232 (11)	0.0595 (5)	
H1A	0.514 (4)	0.723 (3)	0.513 (2)	0.095 (10)*	

### Atomic displacement parameters (Å^2^)

**Table:**

	*U*^11^	*U*^22^	*U*^33^	*U*^12^	*U*^13^	*U*^23^
C1	0.0455 (12)	0.0509 (13)	0.0581 (14)	0.0135 (10)	0.0020 (10)	0.0073 (10)
C2	0.0407 (11)	0.0461 (12)	0.0531 (13)	0.0101 (9)	0.0025 (9)	0.0144 (10)
C3	0.0502 (13)	0.0544 (14)	0.0450 (12)	0.0042 (10)	0.0049 (9)	0.0162 (10)
C4	0.0473 (13)	0.0828 (17)	0.0515 (14)	−0.0030 (12)	−0.0030 (10)	0.0225 (12)
C5	0.0355 (12)	0.097 (2)	0.0641 (16)	0.0140 (12)	0.0045 (10)	0.0258 (14)
C6	0.0472 (13)	0.0812 (17)	0.0544 (14)	0.0032 (12)	0.0098 (11)	0.0187 (12)
C7	0.0554 (14)	0.0666 (15)	0.0481 (13)	0.0146 (11)	0.0067 (10)	0.0136 (11)
C8	0.0451 (12)	0.0563 (13)	0.0467 (12)	0.0123 (10)	0.0005 (9)	0.0063 (10)
C9	0.0540 (14)	0.0765 (17)	0.0651 (16)	0.0282 (12)	0.0135 (12)	0.0193 (13)
C10	0.0403 (12)	0.0554 (13)	0.0621 (14)	0.0109 (10)	0.0007 (10)	0.0158 (11)
C11	0.0336 (11)	0.0555 (13)	0.0607 (14)	0.0095 (9)	0.0008 (9)	0.0145 (11)
C12	0.0512 (14)	0.0622 (16)	0.090 (2)	−0.0012 (12)	−0.0058 (13)	0.0210 (14)
C13	0.0624 (19)	0.112 (3)	0.140 (4)	−0.0064 (18)	0.017 (2)	0.070 (3)
C14	0.071 (2)	0.185 (5)	0.089 (3)	0.037 (3)	0.0249 (19)	0.061 (3)
C15	0.0661 (19)	0.144 (3)	0.067 (2)	0.039 (2)	0.0154 (15)	0.016 (2)
C16	0.0492 (14)	0.0706 (16)	0.0715 (18)	0.0159 (12)	0.0008 (12)	0.0013 (14)
C17	0.115 (3)	0.070 (2)	0.141 (3)	−0.0095 (19)	−0.013 (2)	0.001 (2)
C18	0.0427 (12)	0.0555 (15)	0.0689 (17)	0.0116 (10)	0.0055 (11)	0.0248 (12)
C19	0.086 (2)	0.089 (2)	0.084 (2)	0.0114 (16)	0.0068 (16)	0.0532 (18)
C20	0.140 (3)	0.108 (3)	0.063 (2)	0.007 (2)	0.002 (2)	0.0350 (19)
C21	0.102 (3)	0.162 (4)	0.076 (2)	−0.021 (3)	0.007 (2)	0.028 (2)
C22	0.151 (4)	0.189 (5)	0.110 (3)	0.092 (4)	0.062 (3)	0.059 (3)
C23	0.172 (4)	0.066 (2)	0.080 (2)	0.015 (2)	0.004 (2)	0.0009 (17)
C24	0.169 (5)	0.129 (4)	0.124 (4)	0.081 (3)	0.043 (3)	0.000 (3)
C25	0.0835 (19)	0.0757 (18)	0.0698 (18)	0.0101 (15)	0.0087 (14)	0.0179 (15)
C26	0.134 (3)	0.157 (4)	0.069 (2)	0.027 (3)	−0.003 (2)	0.035 (2)
N1	0.0673 (16)	0.112 (2)	0.0616 (14)	−0.0132 (14)	0.0121 (12)	0.0218 (14)
N2	0.0590 (12)	0.0628 (13)	0.0491 (12)	0.0116 (10)	−0.0013 (9)	0.0009 (10)
N3	0.0744 (18)	0.112 (2)	0.097 (2)	0.0466 (17)	0.0251 (16)	0.0115 (18)
N4	0.0697 (14)	0.0672 (14)	0.0525 (13)	0.0033 (11)	−0.0024 (11)	0.0083 (10)
O1	0.0712 (12)	0.0495 (10)	0.0882 (13)	0.0030 (8)	0.0177 (10)	0.0084 (9)
O2	0.0651 (14)	0.168 (2)	0.0964 (17)	−0.0373 (15)	0.0001 (12)	0.0372 (16)
O3	0.1073 (18)	0.1152 (18)	0.0907 (16)	−0.0244 (14)	0.0072 (13)	0.0478 (15)
O4	0.0826 (13)	0.0792 (13)	0.0575 (11)	0.0114 (10)	−0.0186 (9)	0.0151 (9)
O5	0.0713 (12)	0.0704 (12)	0.0649 (12)	−0.0075 (9)	−0.0074 (9)	−0.0011 (9)
O6	0.136 (3)	0.192 (4)	0.211 (4)	0.007 (3)	0.071 (3)	−0.104 (3)
O7	0.115 (2)	0.155 (2)	0.114 (2)	0.0553 (18)	0.0610 (17)	0.0409 (18)
O8	0.0982 (15)	0.0547 (12)	0.0933 (15)	0.0237 (10)	0.0076 (11)	0.0260 (10)
O9	0.0564 (10)	0.0650 (10)	0.0585 (11)	0.0075 (8)	0.0008 (8)	0.0295 (9)

### Geometric parameters (Å, °)

**Table:**

C1—C8	1.501 (3)	C17—H17C	0.9600
C1—C9	1.532 (3)	C18—O8	1.205 (3)
C1—C2	1.552 (3)	C18—O9	1.312 (3)
C1—H1	0.9800	C19—C20	1.451 (5)
C2—C18	1.530 (3)	C19—O9	1.460 (3)
C2—C3	1.541 (3)	C19—H19A	0.9700
C2—C10	1.561 (3)	C19—H19B	0.9700
C3—O1	1.200 (3)	C20—H20A	0.9600
C3—C4	1.504 (3)	C20—H20B	0.9600
C4—C5	1.541 (4)	C20—H20C	0.9600
C4—H4A	0.9700	C21—N4	1.503 (5)
C4—H4B	0.9700	C21—C22	1.519 (6)
C5—C6	1.492 (4)	C21—H21A	0.9700
C5—C9	1.515 (4)	C21—H21B	0.9700
C5—H5	0.9800	C22—H22A	0.9600
C6—C7	1.352 (3)	C22—H22B	0.9600
C6—N1	1.417 (4)	C22—H22C	0.9600
C7—C8	1.391 (3)	C23—N4	1.482 (4)
C7—H7	0.9300	C23—C24	1.493 (6)
C8—N2	1.364 (3)	C23—H23A	0.9700
C9—N3	1.522 (4)	C23—H23B	0.9700
C9—H9	0.9800	C24—H24A	0.9600
C10—C11	1.502 (3)	C24—H24B	0.9600
C10—H10A	0.9700	C24—H24C	0.9600
C10—H10B	0.9700	C25—N4	1.494 (4)
C11—C12	1.388 (3)	C25—C26	1.496 (5)
C11—C16	1.395 (4)	C25—H25A	0.9700
C12—C13	1.403 (5)	C25—H25B	0.9700
C12—C17	1.500 (5)	C26—H26A	0.9600
C13—C14	1.381 (6)	C26—H26B	0.9600
C13—H13	0.9300	C26—H26C	0.9600
C14—C15	1.330 (6)	N1—O3	1.228 (3)
C14—H14	0.9300	N1—O2	1.234 (3)
C15—C16	1.350 (5)	N2—O5	1.257 (3)
C15—H15	0.9300	N2—O4	1.282 (3)
C16—H16	0.9300	N3—O6	1.183 (4)
C17—H17A	0.9600	N3—O7	1.192 (4)
C17—H17B	0.9600	N4—H1A	0.97 (4)
			
C8—C1—C9	107.6 (2)	H17A—C17—H17C	109.5
C8—C1—C2	112.97 (17)	H17B—C17—H17C	109.5
C9—C1—C2	108.35 (19)	O8—C18—O9	124.8 (2)
C8—C1—H1	109.3	O8—C18—C2	123.7 (2)
C9—C1—H1	109.3	O9—C18—C2	111.5 (2)
C2—C1—H1	109.3	C20—C19—O9	108.8 (3)
C18—C2—C3	109.13 (18)	C20—C19—H19A	109.9
C18—C2—C1	108.56 (18)	O9—C19—H19A	109.9
C3—C2—C1	108.86 (18)	C20—C19—H19B	109.9
C18—C2—C10	108.64 (17)	O9—C19—H19B	109.9
C3—C2—C10	111.77 (18)	H19A—C19—H19B	108.3
C1—C2—C10	109.82 (18)	C19—C20—H20A	109.5
O1—C3—C4	122.3 (2)	C19—C20—H20B	109.5
O1—C3—C2	122.0 (2)	H20A—C20—H20B	109.5
C4—C3—C2	115.6 (2)	C19—C20—H20C	109.5
C3—C4—C5	110.53 (19)	H20A—C20—H20C	109.5
C3—C4—H4A	109.5	H20B—C20—H20C	109.5
C5—C4—H4A	109.5	N4—C21—C22	112.3 (4)
C3—C4—H4B	109.5	N4—C21—H21A	109.1
C5—C4—H4B	109.5	C22—C21—H21A	109.2
H4A—C4—H4B	108.1	N4—C21—H21B	109.1
C6—C5—C9	109.4 (2)	C22—C21—H21B	109.1
C6—C5—C4	111.0 (2)	H21A—C21—H21B	107.9
C9—C5—C4	107.7 (2)	C21—C22—H22A	109.5
C6—C5—H5	109.6	C21—C22—H22B	109.5
C9—C5—H5	109.6	H22A—C22—H22B	109.5
C4—C5—H5	109.6	C21—C22—H22C	109.5
C7—C6—N1	119.4 (2)	H22A—C22—H22C	109.5
C7—C6—C5	121.8 (2)	H22B—C22—H22C	109.5
N1—C6—C5	118.7 (2)	N4—C23—C24	112.9 (3)
C6—C7—C8	121.2 (2)	N4—C23—H23A	109.0
C6—C7—H7	119.4	C24—C23—H23A	109.0
C8—C7—H7	119.4	N4—C23—H23B	109.0
N2—C8—C7	118.8 (2)	C24—C23—H23B	109.0
N2—C8—C1	119.8 (2)	H23A—C23—H23B	107.8
C7—C8—C1	121.1 (2)	C23—C24—H24A	109.5
C5—C9—N3	112.2 (2)	C23—C24—H24B	109.5
C5—C9—C1	110.36 (19)	H24A—C24—H24B	109.5
N3—C9—C1	109.2 (2)	C23—C24—H24C	109.5
C5—C9—H9	108.3	H24A—C24—H24C	109.5
N3—C9—H9	108.3	H24B—C24—H24C	109.5
C1—C9—H9	108.3	N4—C25—C26	114.3 (3)
C11—C10—C2	116.14 (18)	N4—C25—H25A	108.7
C11—C10—H10A	108.3	C26—C25—H25A	108.7
C2—C10—H10A	108.3	N4—C25—H25B	108.7
C11—C10—H10B	108.3	C26—C25—H25B	108.7
C2—C10—H10B	108.3	H25A—C25—H25B	107.6
H10A—C10—H10B	107.4	C25—C26—H26A	109.5
C12—C11—C16	118.1 (2)	C25—C26—H26B	109.5
C12—C11—C10	121.8 (2)	H26A—C26—H26B	109.5
C16—C11—C10	120.1 (2)	C25—C26—H26C	109.5
C11—C12—C13	117.7 (3)	H26A—C26—H26C	109.5
C11—C12—C17	122.4 (3)	H26B—C26—H26C	109.5
C13—C12—C17	119.9 (3)	O3—N1—O2	121.7 (3)
C14—C13—C12	121.3 (3)	O3—N1—C6	120.3 (2)
C14—C13—H13	119.3	O2—N1—C6	118.0 (3)
C12—C13—H13	119.3	O5—N2—O4	119.20 (19)
C15—C14—C13	120.2 (3)	O5—N2—C8	121.4 (2)
C15—C14—H14	119.9	O4—N2—C8	119.4 (2)
C13—C14—H14	119.9	O6—N3—O7	123.5 (4)
C14—C15—C16	119.8 (4)	O6—N3—C9	118.0 (3)
C14—C15—H15	120.1	O7—N3—C9	118.4 (3)
C16—C15—H15	120.1	C23—N4—C25	113.9 (3)
C15—C16—C11	122.8 (3)	C23—N4—C21	111.2 (3)
C15—C16—H16	118.6	C25—N4—C21	113.6 (3)
C11—C16—H16	118.6	C23—N4—H1A	109 (2)
C12—C17—H17A	109.5	C25—N4—H1A	102.1 (19)
C12—C17—H17B	109.5	C21—N4—H1A	106.2 (19)
H17A—C17—H17B	109.5	C18—O9—C19	116.3 (2)
C12—C17—H17C	109.5		
			
C8—C1—C2—C18	177.41 (18)	C2—C10—C11—C16	81.7 (3)
C9—C1—C2—C18	−63.5 (2)	C16—C11—C12—C13	−0.5 (4)
C8—C1—C2—C3	−63.9 (2)	C10—C11—C12—C13	179.8 (2)
C9—C1—C2—C3	55.2 (2)	C16—C11—C12—C17	−179.4 (3)
C8—C1—C2—C10	58.8 (2)	C10—C11—C12—C17	0.9 (4)
C9—C1—C2—C10	177.86 (18)	C11—C12—C13—C14	−0.2 (5)
C18—C2—C3—O1	−116.9 (2)	C17—C12—C13—C14	178.7 (3)
C1—C2—C3—O1	124.8 (2)	C12—C13—C14—C15	0.7 (5)
C10—C2—C3—O1	3.3 (3)	C13—C14—C15—C16	−0.3 (5)
C18—C2—C3—C4	66.9 (2)	C14—C15—C16—C11	−0.5 (4)
C1—C2—C3—C4	−51.4 (2)	C12—C11—C16—C15	0.9 (4)
C10—C2—C3—C4	−172.89 (18)	C10—C11—C16—C15	−179.4 (2)
O1—C3—C4—C5	−123.8 (3)	C3—C2—C18—O8	−150.3 (2)
C2—C3—C4—C5	52.4 (3)	C1—C2—C18—O8	−31.8 (3)
C3—C4—C5—C6	63.0 (3)	C10—C2—C18—O8	87.6 (3)
C3—C4—C5—C9	−56.7 (3)	C3—C2—C18—O9	31.3 (2)
C9—C5—C6—C7	27.1 (3)	C1—C2—C18—O9	149.86 (19)
C4—C5—C6—C7	−91.7 (3)	C10—C2—C18—O9	−90.7 (2)
C9—C5—C6—N1	−151.1 (2)	C7—C6—N1—O3	4.6 (4)
C4—C5—C6—N1	90.1 (3)	C5—C6—N1—O3	−177.1 (3)
N1—C6—C7—C8	178.2 (2)	C7—C6—N1—O2	−175.9 (3)
C5—C6—C7—C8	0.0 (4)	C5—C6—N1—O2	2.4 (4)
C6—C7—C8—N2	−170.9 (2)	C7—C8—N2—O5	171.1 (2)
C6—C7—C8—C1	2.6 (4)	C1—C8—N2—O5	−2.4 (3)
C9—C1—C8—N2	142.2 (2)	C7—C8—N2—O4	−8.0 (3)
C2—C1—C8—N2	−98.2 (2)	C1—C8—N2—O4	178.4 (2)
C9—C1—C8—C7	−31.2 (3)	C5—C9—N3—O6	−162.6 (4)
C2—C1—C8—C7	88.3 (3)	C1—C9—N3—O6	−40.0 (4)
C6—C5—C9—N3	66.1 (3)	C5—C9—N3—O7	20.2 (4)
C4—C5—C9—N3	−173.18 (19)	C1—C9—N3—O7	142.8 (3)
C6—C5—C9—C1	−55.9 (3)	C24—C23—N4—C25	−63.4 (4)
C4—C5—C9—C1	64.9 (3)	C24—C23—N4—C21	166.6 (3)
C8—C1—C9—C5	57.4 (3)	C26—C25—N4—C23	−65.5 (4)
C2—C1—C9—C5	−65.0 (2)	C26—C25—N4—C21	63.3 (4)
C8—C1—C9—N3	−66.3 (3)	C22—C21—N4—C23	−168.0 (3)
C2—C1—C9—N3	171.2 (2)	C22—C21—N4—C25	61.9 (4)
C18—C2—C10—C11	26.0 (3)	O8—C18—O9—C19	−2.8 (4)
C3—C2—C10—C11	−94.5 (2)	C2—C18—O9—C19	175.5 (2)
C1—C2—C10—C11	144.6 (2)	C20—C19—O9—C18	−159.9 (3)
C2—C10—C11—C12	−98.6 (3)		

### Hydrogen-bond geometry (Å, °)

**Table:**

*D*—H···*A*	*D*—H	H···*A*	*D*···*A*	*D*—H···*A*
N4—H1A···O4^i^	0.97 (4)	1.78 (4)	2.740 (3)	168 (3)
C17—H17A···O7^ii^	0.96	2.55	3.434 (5)	154
C10—H10B···O5	0.97	2.43	3.244 (3)	142
C17—H17C···O8	0.96	2.51	3.320 (5)	142
C25—H25A···O4	0.97	2.58	3.516 (4)	164

Symmetry codes: (i) −*x*+1, −*y*+1, −*z*+1; (ii) *x*−1, *y*, *z*.
